# Cross-cultural adaptation, reliability and validity of the Turkish version ofPatient-Specific Functional Scale in patients with chronic neck pain

**DOI:** 10.3906/sag-1905-91

**Published:** 2020-06-23

**Authors:** Gamze YALÇINKAYA, Bilge KARA, Mehmet Nuri ARDA

**Affiliations:** 1 Institute of Health Sciences, Dokuz Eylül University, İzmir Turkey; 2 School of Physical Therapy and Rehabilitation, Faculty of Health Sciences, Dokuz Eylül University, İzmir Turkey; 3 Department of Neurosurgery, Faculty of Medicine, Dokuz Eylül University, İzmir Turkey

**Keywords:** Outcome measures, disability, neck pain, reliability, validity

## Abstract

**Background/aim:**

Current clinical guidelines recommend to use both clinical and self-reported measurements for evaluation of chronic neck pain. Among the self-reported outcomes, Neck disability index and patient-specific functional scale are the most widely used and recommended instruments. The purpose of our study was to determine the test-retest reliability and validity of patient-specific functional scale which was not validated in Turkish language previously.

**Materials and methods:**

Translation and adaptation process had conducted according to the Beaton et al. Sociodemographic data, Turkish version of patient-specific functional scale and neck disability index were recorded at the initial assessment. Retest assessment was produced for reliability analyses and intraclass correlation coefficient (ICC3,2) was determined. The correlations between patient-specific functional scale and neck disability index and hypothesis testing were examined for the convergent and construct validity analysis.

**Results:**

The final form was completed by 110 chronic neck pain patients (Male: 33; mean ages: 43.13 ± 13.75 years, Female: 77; mean ages: 44.45 ± 14.38). Test-retest reliability of patient-specific functional scale was found good level (ICC: 0.85). The relationship between patient-specific functional scale and neck disability index was found moderate level (P < 0.05, rho: –0.578). The median score of PSFS-T in the low disability group was significantly higher than the high disability group in the hypothesis testing of construct validity (P < 0.001).

**Conclusion:**

The Turkish version of the patient-specific functional scale is a valid and reliable scale for evaluating functional status in patients with chronic neck pain.

## 1. Introduction

Neck pain is a widespread problem which affects between 30%–50% of general population in a certain period of their lives [1]. Female gender, older age, high job demands, smoking history, low social/work support and prior history of low back pain were reported as risk factors of chronic neck pain [2–4]. Neck pain patients suffer from recurrent pain and this process is commonly become chronic. The latest recommendations of the International Association for the Study of Pain about the management of chronic pain has been highlighted the importance of patient-specific self-reports during the evaluation [5]. Moreover, Turk et al. has been reviewed that biopsychosocial and behavioral factors are the key points for the assessment of the chronic pain. Therefore, rehabilitation assessments seem to be shifting from traditional evaluations to a more holistic approach. Determining the patient-specific goals and making the patient part of the treatment process is very essential for the management of chronic pain [2,5,6].

There are several relevant questionnaires in current literature for evaluating the pain and the disability associated with the neck pain. The neck outcome score, the fremantle neck awareness questionnaire, the Copenhagen neck functional disability scale,u2929, and neck disability index were translated and validated before into Turkish language [7–11]. However, the current guidelines and systematic reviews have mostly recommended neck disability index (NDI) and the patient-specific functional scale (PSFS) in the assessment process [2,6]. 

The patient specific functional scale (PSFS) has been developed by Stratford et al. for determined the functional ability of patients with musculoskeletal chronic pain [12,13]. PSFS is a self-administered scale that the patient lists the activities of difficult to attend and score them in the goal setting process [14]. The examiner records the scores according to assessment date and in the rehabilitation process, patients have opportunities to observe the improvement oftheir limited activities in daily life. PSFS is short, time-consuming and easy to use scale and it has been reported in the literature as valid, reliable and responsive in terms of psychometric properties for different musculoskeletal conditions such as low back pain, carpometacarpal osteoarthritis and lateral epicondylitis [15]. All these properties of PSFS provide advantageous in clinical management. Besides, it has been used many randomized controlled trials as an outcome measure [16–21]. PSFS is also valid and reliable for chronic neck pain patients. 

The availability of the validation of a recommended questionnaire in another language and culture is commonly required to be used [22,23]. While the NDI has been validated in the Turkish language before, as to our knowledge no attempt has been made for the validation of PSFS.PSFS has been validated in Finnish, Swedish, Portuguese, Japanese, Nepali, and Dutch [24–29]. The aim of this study is to conduct the test-retest reliability and convergent-construct validity of the Turkish version of PSFS in neck pain patients.

## 2. Materials and methods

This validation study was conducted in the School of Physical Therapy and Rehabilitation of Dokuz Eylül University between October 2016 and April 2017. The ethical approval was obtained from Noninvasive Research Ethics Committee of Dokuz Eylül University (No: 2016/ 25-15, Protocol Number: 2930, Date: 22.09.2016) prior to the study and all procedures were conducted according to the Declaration of Helsinki. The signed informed consents were obtained from all participants prior to the study. The required permission has been obtained from the original author of the scale (Paul Stratford) via e-mail.

### 2.1. Patients

The sample of the study was the patients with chronic neck pain complaints. The inclusion criteria were determined as following: the ability to read and understand Turkish and having a chronic neck pain for at least three months. Exclusion criteria were patients with red flag medical conditions (tumors, vertebral fractures, traumatic injuries etc.), cervical radiculopathy signs, having psychiatric disorders and those who having undergone spinal surgery, an ongoing physical therapy program and could not read in Turkish language. The physiotherapist informed the patients about the study and their informed consent forms were obtained.

### 2.2. Translation and cultural adaptation of the patient specific functional scale

The Turkish version of the PSFS (PSFS-T, Appendix 1) was constructed by a repeated back and forward translation process. The process was managed by an independent translator team with following the translation and cultural adaptation processes as described by Beaton et al. (Table 1) [22].

**Table 1:** Translation and cultural adaptation process.

Translation and cultural adaptation process	Preparation	Permission for the translation and cultural adaptation of PSFS was obtained via e-mail from Prof. Paul Stratford who developed original scale.
	First Step	Forward translation process was performed by 2 independent translators whose main language is the target language and who can speak fluently in both languages.
	Second Step	The target and independent translations were combined.
	Third Step	Backward translation process was carried out by 2 independent translators whose main language is the source language and who can speak fluently in both languages.
	Fourth Step	Backward translation was evaluated to make sure concept equality was provided. Then, all the translations and the source version were integrated.
	Fifth Step	PSFS-T was performed by 10 people with neck pain to assess the clarity and completeness of the survey questions.
	Sixth Step	It was decided the PSFS-T were quite understandable and had no uncertainty on the target population. The final version was achieved to be used for the study.

### 2.3. Assessments

#### 2.3.1. Neck disability index (NDI)

NDI is a widely used self-report questionnaire to assess the symptoms of neck pain patients and the limitations of their functional activities. The questionnaire had 10 sections; pain intensity, personal care, lifting, reading, headaches, concentration, work, driving, sleeping, and recreation. Each item scored between 0 (no disability) and 5 (total disability). NDI was reported as a valid and reliable tool for evaluating neck symptoms and functions according to the current literature and guidelines. The Turkish version of the NDI was used, and the validation was performed by Aslan et al. in 2008 [11]. 

#### 2.3.2. The patient specific functional scale (PSFS)

PSFS was developed by Stratford et. al for evaluating patient-specific functional disability level and have a good reliability and validity [13]. Patients were asked to list three activities which cause the most difficulty related to their neck pain. Then, each activity was scored between 0 (unable to perform activity) and 10 (able to perform activity at the same level as before the onset of symptoms) [12]. 

### 2.4. Statistical methods

Analyzes of data were performed by using “SPSS 20.0 for Windows” program. The cultural adaptation of the PSFS-T was evaluated at the beginning of the study (Table 1). Sample size was determined as 83 chronic neck pain patients by calculation in GPower 3.1 program using the data of PSFS Japanese version study convergent validity data [25]. And, the study was completed with 110 patients (77 women, 33 men). Normal distribution was evaluated with Kolmogorov-Smirnov test. However, nonparametric analyses were used since there was no compatibility with normal distribution.

#### 2.4.1. Reliability 

In order to determine the test-retest reliability intraclass correlation coefficient (ICC) was calculated. PSFS-T was reapplied to the first 30 patients 4–14 days following the initial evaluation [30]. The (ICC3,2) model was used. Level of ICC was interpreted using following criteria: <0.5 = weak, 0.5–0.75 = moderate, 0.75–0.90 = good, >0.9 = excellent [31].

#### 2.4.2. Convergent and construct validity

Convergent validity analysis was determined by performing the Spearman correlation analysis of PSFS-T and NDI due to nonparametric conditions. The level of correlation was interpreted as 0–0.25: no relationship, 0.25–0.50: fair relationship, 0.5–0.75: good relationship, >0.75: excellent relationship [32]. Therefore, there was a excellent relationship between NDI and PSFS-T in the hypothesis one as these instruments are based on a parallel construct. 

Construct validation by extreme groups (known group validity) is a type of validation where the instrument is assessed on two extreme groups, which should score significantly different on the measurement instrument [33]. Extreme groups were defined on initial disability levels by NDI. We assumed that patients with high disability (>15) would have a higher level of perceived disability on PSFS. The Mann Whitney-U test was used to test the difference between known groups. For hypothesis 2, we expected a significant difference between the groups (high and low disability) according to PSFS.

## 3. Results

A total of 110 chronic neck pain patients included in this study. Descriptive characteristics of patients and disability scores related to NDI and PSFS-T scales were summarized in Table 2. 

**Table 2:** Descriptive characteristics of patients (n: 110).

Variable	Value [mean ± SD, n (%)]
Age (year)	44.1 ± 14.1
Weight (kg)	72.1 ± 12.8
Height (cm)	166.6 ± 9
BMI (kg/m2)	25.9 ± 4
Male [n (%)]	33 (30%)
Female [n (%)]	77 (70%)
Pain duration (month)	43.2 ± 49.5
PSFS-T	18.1 ± 4.1
NDI	17.3 ±5.6

SD: Standard deviation; BMI: Body mass index; PSFS-T: Turkish version of patient specific functional scale; NDI: Neck disability index.

### 3.1. Test-retest reliability outcomes of PSFS-T

While the ICC scores for the first (ICC = 0.73) and the second activities (ICC = 0.76) showed moderate reliability in PSFS-T, third activity (ICC = 0.85) and total scores (ICC = 0.85) showed good reliability. Test-retest results, ICC scores, confidence intervals (CI) were summarized in Table 3.

**Table 3:** Test-retest reliability results of PSFS-T.

	Initial evaluation(Mean ± SD)	Retest evaluation(Mean ± SD)	ICC	95% CI
First activity	6.14 ± 1.66	5.97 ± 1.45	0.73	0.44–0.87
Second activity	6.09 ± 1.68	6.37 ± 1.54	0.76	0.51–0.89
Third activity	5.95 ± 1.68	6.17 ± 1.78	0.85	0.68–0.93
PSFS-T total score	18.17 ± 4.14	18.50 ± 3.81	0.85	0.67–0.93
PSFS-T mean score	6.06 ± 1.38	6.17 ± 1.27	0.85	0.67–0.93

SD: Standard deviation; ICC: Intraclass correlation coefficient; 95% CI: 95% confidence interval; PSFS-T: Turkish version of patient specific functional scale.

### 3.2. Convergentand construct validity outcomes of PSFS-T

A moderate and negative correlation was determined between PSFS-T and NDI (rho = –0.578, P < 0.01). When the patients were examined according to the activities they reported in the first place, the correlation increased to an excellent level (r = –0.865) for reading, however, cleaning (r = –0.487) and lifting a thing over the head (r = –0.575) activities showed moderate correlations (Table 4). In this context, hypothesis one was not defined, as the relationship between PSFS-T and NDI was –0.578, indicating a good relationship instead of a excellent relationship (>0.75).

**Table 4:** Correlations between NDI and PSFS-T Scores.

	n	rs / rp	P
Total score	110	-0.578s	<0.001*
Reading	25	-0.865p	<0.001*
Cleaning up	22	-0.487p	<0.001*
Lifting	15	-0.575p	<0.001*

rs: Spearman correlation coefficient; rp: Pearson correlation coefficient; *: P < 0.05.

Hypothesis 2 was confirmed as differences between “known groups” were statistically significant. The median score of PSFS-T in the low disability group was significantly higher than the high disability group (P < 0.001) (Table 5).

**Table 5:** Differences between Low and High Disability Groups According to PSFS-T.

	Low disability (n = 44)(NDI ≤ 15) Median (Q1–Q3)	High disability(n = 66)(NDI > 15) Median (Q1–Q3)	P
PSFS-T	7 (6–7.67)	5.67 (5–6.42)	<0.001*

NDI: Neck disability index; Q1: First quartile (25%); Q3: Third quartile (75%); PSFS-T: Patient-specific functional scale Turkish version; *: Mann Whitney-U Test, P < 0.05.

### 3.3. Activities with limited participation according to PSFS-T results 

As the PSFS is a personalized questionnaire, chronic neck pain patients reported difficulties in 27 different activities. The 3 most frequently reported activities were reading books (19.7%), cleaning (18.1%) and lifting a thing over the head (12.4%) (Table 6). 

**Table 6:** Activities with limited participation according to patient-specific functional scale.

Activities	Reporting percentages %
Reading book Cleaning Lifting a thing over the head Watching television Driving car Using computer Making crafts Gardening Praying Cooking Doing sport Using mobile phone Wearing Studying lesson Walking Hanging curtain Taking shower Reaching out an object Tying shoes Carrying hand bag Traveling Combing hair Shopping Painting wall Painting Writing Doing Puzzle	19.7 18.1 12.4 7.2 5.7 4.83.63.63.02.12.42.42.12.4 1.8 1.5 0.9 1.2 0.6 0.6 0.6 0.6 0.3 0.3 0.3 0.6 0.6

## 4. Discussion

The importance of evaluating functional activity limitations with reliable and valid tools is increasing day by day in physiotherapy [34]. These outcome measures help to determine the benefits of treatment and allow us to follow the changes in the patient’s conditions. However, mostly, other clinical methods such as muscle strength measurement, the range of motion evaluation and pain assessment are performed in musculoskeletal physiotherapy practice [35,36]. Current clinical guidelines related to physiotherapy assessments in neck pain recommend including functional activity and participation assessments during the evaluation. In this manner, PSFS is a widely recommended tool [2,6,37]. However, in the light of the current literature, the PSFS scale has not been found translated into Turkish before. Thus, PSFS was adapted in Turkish language and found valid and reliable in terms of evaluating functional activities of chronic neck pain patients in the present study. 

As to our knowledge, there are 6 studies focus on reliability and validity of PSFS in patients with neck pain up to date [13,25,29,38–40]. A comparison of the previous studies and the recent study was provided in Table 7. The major part of these studies was conducted in English speaking countries except the Japanese and Dutch version studies [25,29]. The studies were conducted in different neck pain conditions such as radiculopathy, neck dysfunction, and chronic neck pain. Most of the studies reported high test-retest values (ICCs: between 0.82–0.98) as we determined in the present study (ICC: 0.85). However, Young et al. reported very low test-retest value (ICC: 0.17) for the PSFS. These authors concluded that ICC scores may be affected by dynamic symptom distribution of cervical radiculopathy patients [13,25,38–40]. 

**Table 7:** Comparison of current study results with previous studies.

Author/Country/YP	SS	Reliability (ICC)	Validity tests	rs/ rp
Westaway/Canada/1998	31	0.92	P-NDI	0.58p
Cleland/USA/2006	38	0.82	P-NPRS	0.80p
Young/Canada/2010	165	0.17	N/A	N/A
Abbott/New Zeeland/2014	98	N/A	S-NDI	–0.56s
Nakamaru/Japan/2015	103	0.98	P-NDI	–0.35p
De Graaf/Netherlands/2019	100	N/A	P-NDI	0.54s
Yalcinkaya/Turkey/2019	110	0.85	S-NDI	–0.57s

YP: Year of publication; SS: Sample size; ICC: Intraclass correlation coefficient; rs: Spearman correlation coefficient; rp: Pearson correlation coefficient; P: Pearson’s correlation test; NDI: Neck disability index; NPRS: Numeric pain rating scale; N/A: Not applicable; S: Spearman’s correlation test.

The present the validity analysis of the PSFS-T were compatible with Japanese and Dutch version studies within the scope of convergent and construct validity [25,29]. Nakamaru et al. found low relationship between NDI and PSFS in the convergent analysis (r: –0.35) (25). However, we determined moderate relationship between NDI and PSFS (rho: –0.57), similary with Dutch version study (rho: 0.54). We thought that the differences in the correlation results could be related with cultural factors since the sample sizes were similar of the compared studies. Besides, Thoomes-de Graaf et al. was indicated a significant difference between low pain and high pain groups for construct validity hypothesis of PSFS [29]. In this context, our result was similar with the Dutch version. We also found a significant difference between low and high disability groups in accordance to PSFS-T for the hypothesis of construct validity. Future studies can also be carried out on the sensitivity analysis of PSFS-T which recommended on assessing measurement properties in the current literature [33].

Cleland et al. listed the most reported activities in PSFS as driving car (50%), sleeping (50%) and using the computer (40%) respectively [32]. In our study, reading (19.7%), cleaning (18.1%) and carrying heavy things (12.4%) were reported as the hardest activities related to neck pain, respectively [38]. The nature of PSFS is a self-administered and different cultures or living styles could change the affected activities reasonably. Additionally, if a patient’s activity selection on PSFS matched with NDI activities, correlation coefficient could vary. In our study, the correlation between the total PSFS and NDI scores was excellent in patients who listed “reading” activity in the first place (r: –0.865). In contrast, the correlation between the total PSFS and NDI scores was fair in patients who listed “cleaning” activity which is not covered by NDI (r: –0.487). Thus, we think that reported activities in PSFS scale might conduce different correlations with NDI total scores (Table 4). 

Neck pain does not only lead a decline in physical functioning but also causes additional negative emotional conditions such as depression and fear avoidance beliefs [41]. Therefore, assessing the restriction in the functional activities of the patients who experience chronic problems might lead the health professionals to offer more reliable outcome measures. In this direction, the use of self-reported outcome measures in physiotherapy is getting increase [14,36,37]. However, a survey study among the physiotherapists showed that self-reported measurements are not preferably due to time constraints, the length of the scales and long duration of appropriate scale selection [35]. In this manner, PSFS is a very available scale for musculoskeletal evaluation, as it is short and does not contain too many questions. Therefore, PSFS might help to clinician cover the symptoms of the patient more in detail [37].

It seems logical to use a tool which serves specifically to a population. However, tools that are specific to a disease or condition could not cover the needs of all populations with the same level of sensitivity. For instance, a tool which is specific to sedentary populations might not provide accurate results in the athletic population. However, PSFS is a person specific tool which allows collecting results unique to the patient. In the report of Fairbairn et al. 2911 different activity items which were collected via PSFS and were found 100% matched with the international classification of functioning disability and health (ICF) [42]. These results indicate that PSFS might be able to cover the ICF which aims to build a common language system for health. Moreover, PSFS was used in a variety of musculoskeletal conditions such as lateral epicondylitis, upper extremity injuries, osteoarthritis, low back pain [15]. Future studies could be conducted about its validity and reliability in the other musculoskeletal conditions. 

Besides all the patient specific features of the PSFS, the use of this tool for academic purposes might be a challenge. Wiitavaara et al. performed a systemic review on shoulder-neck pain related outcome tools and mentioned that a comprehensive assessment should include pain, physical condition, mental and cognitive situation assessments. They also stated that PSFS is a really sensitive scale for patient follow-up, but the analysis of the scale is so difficult especially comparing the patients’ conditions to each other[43]. Similarly, Pietrobon et al. reviewed all neck pain scales and recommended 5 outcome scales including PSFS and they concluded same as in Wiitavaara’s report [44]. According to both authors, PSFS is a very patient specific scale and useful in clinical settings, but it is also very hard to use the PSFS in research studies.

The Turkish version of PSFS was found valid and reliable for Turkish-speaking neck pain patients in the present study. However, no follow-up periods were provided. Therefore, responsiveness analyses were not discussed. This is the limitation of our study. 

## Acknowledgments/Conflict of interest

The authors would like to thank Paul Stratford for their permission to conduct this study, translators for their voluntariness and patients for their participation. Also, the authors have appreciated to Deniz Bayraktar on their help in language corrections. All authors declare that there is no conflicts of interest.

## Informed consent

The ethical approval was obtained from Noninvasive Research Ethics Committee of Dokuz Eylül University (No: 2016/ 25-15, Protocol Number: 2930, Date: 22.09.2016) prior to the study and all procedures were conducted according to the Declaration of Helsinki. The signed informed consents were obtained from all participants prior to the study.

## Appendix 1. Patient-specific functional scale Turkish version

**Table A1:** Skala klinisyen tarafından hastaya okunur ve doldurulur. Hikâye alımının sonunda ve fizik muayeneden önce tamamlanır. Puanlama Şeması (Hastaya skalayı gösterin)

0	1	2	3	4	5	6	7	8	9	10
										

0: Aktiviteyi yapamayacak durumda olmak 10: Boyun ağrısı başlamadan önceki seviyede aktiviteyi yapabiliyor olmak

**Table:** A2

Aktivite	Tarih/Puan	Tarih/Puan	Tarih/Puan	Tarih/Puan	Tarih/Puan
1					
2					
3					

© 1995, P Stratford, reprinted with permission.
